# Simultaneous meningioma and brain metastasis from renal cell carcinoma - a rare presentation. Case report: Meningioma e metástase cerebral de carcinoma de células renais simultâneos - uma apresentação rara. Relato de caso

**DOI:** 10.1590/1516-3180.2016.016228102016

**Published:** 2017-05-29

**Authors:** Aline Lariessy Campos Paiva, João Luiz Vitorino Araujo, Vinicius Ricieri Ferraz, José Carlos Esteves Veiga

**Affiliations:** I MD. Neurosurgery Resident, Faculdade de Ciências Médicas da Santa Casa de São Paulo (FCMSCSP), São Paulo (SP), Brazil.; II PhD. Assistant Neurosurgeon, Faculdade de Ciências Médicas da Santa Casa de São Paulo (FCMSCSP), and Neurosurgeon, Instituto do Câncer Arnaldo Vieira de Carvalho (ICAVC), Oncocenter and Hospital Nove de Julho, São Paulo (SP), Brazil.; III PhD. Full Professor and Head, Discipline of Neurosurgery, Faculdade de Ciências Médicas da Santa Casa de São Paulo (FCMSCSP), São Paulo (SP), Brazil.

**Keywords:** Neoplasm metastasis, Brain neoplasms, Carcinoma, renal cell, Meningioma, Cerebral ventricle neoplasms

## Abstract

**CONTEXT:**

Brain metastases are the most common tumors of the central nervous system. Because of their high frequency, they may be associated with rare situations. Among these are tumor-to-tumor metastasis and an even a rarer situation called simultaneous brain tumors, which are more related to primary tumors of the reproductive and endocrine systems.

**CASE REPORT:**

A 56-year-old male patient with a history of renal cell carcinoma (which had previously been resected) presented with a ventricular lesion (suggestive of metastatic origin) and simultaneous olfactory groove lesion (probably a meningioma). First, only the ventricular lesion was dealt with, but after a year, the meningothelial lesion increased and an occipital lesion appeared. Therefore, both of these were resected in a single operation. All the procedures were performed by the same neurosurgeon. The patient evolved without neurological deficits during the postoperative period. After these two interventions, the patient remained well and was referred for adjuvant treatment.

**CONCLUSIONS:**

This study provides the first description of an association between these two tumors. Brain metastases may be associated with several lesions, and rare presentations such as simultaneity with meningioma should alert neurosurgeons to provide the best oncological treatment.

## RESUMO

**CONTEXTO:**

As metástases cerebrais são os tumores mais comuns do sistema nervoso central e, devido à sua elevada frequência, podem estar associadas a situações raras. Entre estas estão as *“tumor to tumor metastasis”* e uma situação ainda mais rara chamada de tumores cerebrais simultâneos, mais relacionados a tumores primários dos sistemas endocrinológico e reprodutivo.

**RELATO DE CASO:**

Um homem de 56 anos com histórico de câncer de células renais (extirpado previamente) apresentou-se com lesão ventricular (sugestiva de origem metastática) e simultaneamente com uma lesão em topografia de goteira olfatória (provavelmente meningioma). Primeiramente, apenas a lesão ventricular foi abordada, porém após um ano, a lesão meningotelial aumentou e uma lesão occipital apareceu e então ambas foram ressecadas em uma única cirurgia. Todos os procedimentos foram realizados pelo mesmo neurocirurgião. O paciente evoluiu sem déficits neurológicos no período pós-operatório. Após essas duas intervenções, o paciente permaneceu bem, sendo encaminhado para tratamento adjuvante.

**CONCLUSÕES:**

O presente trabalho é a primeira descrição da associação encontrada entre esses dois tumores. As metástases cerebrais podem associar-se a várias lesões, e manifestações raras, tais como apresentação simultânea com meningioma, devem alertar o neurocirurgião a fornecer o melhor tratamento oncológico.

## INTRODUCTION

Brain metastases constitute a common complication of advanced primary tumors. Therefore, they are an important issue that guides the approach taken towards patients with a diagnosis of cancer.^1^

The incidence of brain metastases is about 9 to 17%, based on various studies.^1^ However, the exact incidence is thought to be higher, possibly because there are many asymptomatic patients. In several studies, only surgical metastatic disease is included in the statistical analysis.^1^

Brain metastases are observed in 2 to 17% of patients with metastatic renal cell carcinoma (mRCC).^2^^,^^3^ These patients usually require a neurosurgical approach and adjuvant therapies, especially radiotherapy. However, despite optimal treatment, patients presenting with brain metastasis have a very poor prognosis and probably also have other compromised organs. Another factor associated with increased mortality is that mRCC does not have a good response to radiation.^2^^,^^3^^,^^4^

There are two entities that are rarely related to brain metastases but which, when they occur, it is important to be aware of. The first of these is tumor-to-tumor metastasis^5^ (collision tumor is used as a synonym by some authors), which was first described in 1902. This is a well-documented phenomenon in which a host tumor that is usually more indolent serves as the source for growth of a more aggressive neoplasm such as a meningioma, thus leading to growth of a high-grade glioma or metastatic lesion.^6^^,^^7^^,^^8^

The second of these is an even rarer phenomenon that has been named synchronous or simultaneous tumors, and which forms the topic of the present report. These occur when two histological tumors compromise the central nervous system (CNS) at the same time but there is no histopathological evidence that one tumor served as the source of growth for the other, as occurs in the tumor-to-tumor entity.^9^^,^^10^

This report aimed to present a unique case of simultaneous benign meningioma and brain metastasis from renal cell carcinoma in a male adult. We were unable to find any similar cases reported in the literature, through reviewing the MEDLINE database.

## CASE REPORT

A 56-year-old male patient came to our neuro-oncology service in 2013, with a history of mild frontal headache, but without neurological symptoms. He had a history of renal cell carcinoma in his right kidney and had undergone nephrectomy in 2011. In the same year, he underwent follow-up examinations but without evidence of brain metastatic disease. He had no other comorbidities.

**Figure 1.**
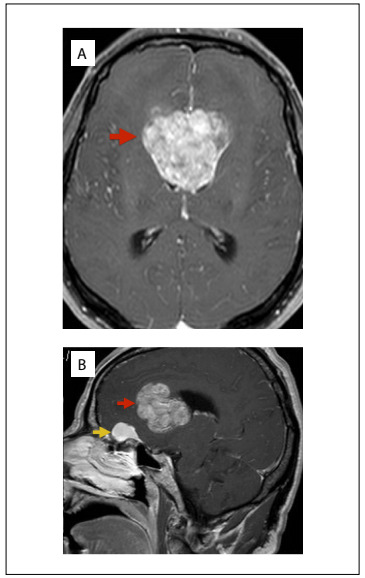
Magnetic resonance imaging of a patient with simultaneous brain tumors: A - axial image showing ventricular tumor; B - sagittal image showing two lesions: olfactory groove meningioma and ventricular tumor.

The headache became progressively worse and was associated with nausea, photophobia and phonophobia. In 2015, on control brain magnetic resonance imaging (MRI), the presence of an intraventricular tumor was noticed (Figure 1), along with another lesion in the olfactory groove (on MRI, it was suggestive of a meningioma). A neurosurgical approach was used to treat the ventricular lesion, consisting of transcallosal tumor resection, which was performed in May 2015. The procedure was free from complications, gross total removal was achieved and the patient reported improvement of the headache. He was referred for neuro-oncology outpatient follow-up and for radiotherapy evaluation. Only the larger of the two lesions was resected on this occasion because two different approaches performed at the same time might have increased the morbidity and, moreover, the patient did not have any neurological deficits at this time.

**Figure 2.**
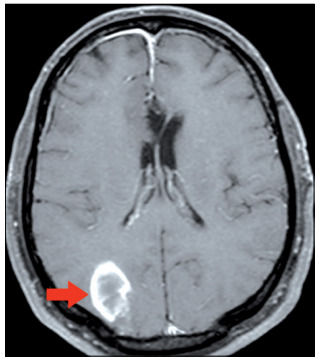
Axial contrasted T1-weighted magnetic resonance imaging exam showing a right occipital lesion after radiation.

Five months after the first procedure, the patient complained of visual impairment (which upon physical examination was found to be due to left hemianopia) and frontotemporal headache. Because of this, MRI was performed again and this revealed another lesion, in the right occipital lobe (Figure 2), probably of metastatic origin. The patient underwent whole-brain radiotherapy at this time, without any surgical indication.

**Figure 3.**
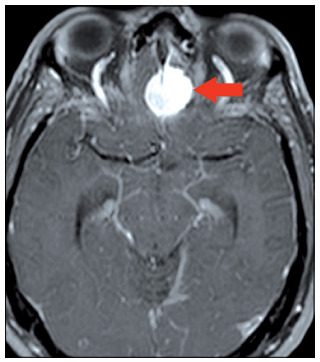
Axial contrasted T1-weighted magnetic resonance imaging exam showing olfactory groove meningioma

The olfactory groove lesion increased after radiation therapy had been completed (Figure 3) and the patient reported that his headache had returned. At this time, neurosurgical resection of the two lesions (the olfactory groove meningioma and the occipital lesion) in a single procedure was proposed. Given the possibility of tumor-to-tumor metastasis, it was very important to determine whether the meningioma had served as a basis for the metastatic lesion, in order to better define the complementary treatment.

**Figure 4.**
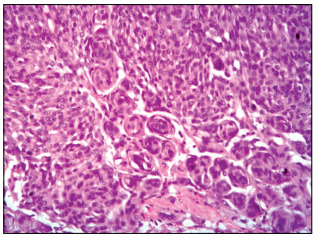
Mature neoplasia of meningothelial origin, characterized by uniform lobed cells. Concentric arrangements are frequent in these tumors. Hematoxylin-eosin staining, 40 x magnification.

**Figure 5.**
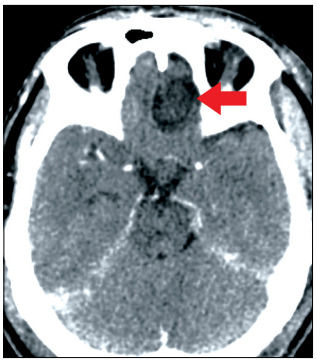
Brain computed tomography showing Simpson II resection of olfactory groove meningioma.

The surgery was performed without complications and the histopathological and immunohistochemical analyses confirmed that the olfactory groove lesion was a grade I meningioma (Figure 4), without evidence of tumors of another origin differing from the meningothelial lineage. The only radionecrosis was observed at histopathological analysis of the occipital lesion. Simpson II resection was performed (Figure 5). These two surgical procedures (one to treat the intraventricular metastasis and the other to deal with the olfactory groove meningioma and the occipital lesion) were performed by the same oncological neurosurgeon (JLVA).

The patient reported improvement of his headache and was discharged for outpatient follow-up with the neurosurgical and neuro-oncology team. Complete screening with the aim of revealing any other metastatic lesion that might have been present was performed, consisting of computed tomography (CT) scans on the chest, abdomen and pelvis. There was no evidence of any local or metastatic recurrence.

## DISCUSSION

Brain metastases constitute one of the most common neurological complications in oncological patients with advanced disease.^1^ In some cases, they may be the initial manifestation, which then leads to diagnosing the primary tumor.^11^ The signs and symptoms are nonspecific and may vary according to the site and size. In rare cases, they may manifest as lesions in the scalp and skull.^12^^,^^13^ The incidence rate is about 9 to 17%, based on various studies, although the exact incidence is thought to be higher.^1^^,^^3^

The blood-brain barrier (BBB) and absence of a lymphatic system are factors that make dissemination of cancerous cells more difficult. Thus, patients with brain metastases generally also have extracranial lesions. This shows that when tumor cells invade brain structures, the disease is more advanced and has a worse prognosis.^2^^,^^3^ Conditions that alter immunological defenses, such as human immunodeficiency virus infection, may be associated with brain metastasis. There are some hypotheses stating that such conditions could favor appearance of some tumors.^14^

Regarding metastatic renal cell carcinoma, brain lesions generally do not occur at the same time as the primary tumor. Some studies have shown that the incidence is highest around 10 to 13 years after the initial nephrectomy.^1^ The treatment may be difficult because central nervous system lesions are usually resistant to chemotherapy and radiotherapy.^15^ Nonetheless, these lesions may respond to immunotherapy using alpha-interferon^16^ or interleukin (IL)-2. After treatment of brain metastases, the median survival is about 4-5 months,^3^ but aggressive surgical resection significantly increases this period.

There are some hypotheses explaining why brain metastasis may appear after a long period, in the absence of other metastatic lesions^16^ in renal cell carcinoma cases. One hypothesis is that this might be because, in the initial stage, the brain metastasis is microscopic and does not cause any neurological symptoms. Another hypothesis is that adjuvant therapy for renal cell carcinoma decreases host immunopotency and thus leads to faster development of brain lesions.^16^ In the present report, the patient evaluated initially did not present central nervous system impairment: it was only after some years that it was found that he had brain metastasis.

After disruption of the BBB, migration of inflammatory cells, including tumor-associated macrophages (TAMs)^17^^,^^18^ may contribute towards persistence of increased vascular permeability. TAMs are recruited to tumors through specific chemokine/ chemokine receptor interactions. When neoplastic cells invade the central nervous system and a metastasis develops, the lesion is seen to be well vascularized and is susceptible to spontaneous intracranial hemorrhage,^4^ which may include intraventricular bleeding. Metastatic renal cell carcinoma has a unique affinity to the ventricular system, in close association with the choroid plexus, probably due to a chemokine cascade.^11^

Multiple primary intracranial tumors of different histological types are rare, except for cases observed after radiotherapy or in situation of phacomatosis^19^ such as Von Recklinghausen syndrome. However, multiple brain tumors in the absence of these conditions constitute an even rarer phenomenon.^10^

When a lesion serves as the source for growth of another neoplasia, this is considered to constitute an entity named tumor-to-tumor metastasis (or collision tumor). A more indolent tumor is generally the substratum for an aggressive lesion.^5^ In the present case, however, the histopathological analysis (Figure 5) did not reveal that one tumor had served for growth of another but, rather, that two different tumors had simultaneous occurrence at different sites.

Because brain metastases have higher incidence than primary central nervous system tumors, they present greater involvement in cases of multiple brain tumors, such as collision tumors or simultaneous tumors. Systemic cancers rarely metastasize into preexisting intracranial neoplasms; meningiomas are the major recipient of these metastases.^8^

Neuroimaging is unable to predict which entity was present. Only when accurate histopathological and immunohistochemical analysis is performed is it possible to confirm whether one tumor has served as source of growth for another or whether the observed tumor represents two different lesions occurring simultaneously. Brain metastases can sometimes behave on CT and MRI as images of typical meningiomas and thus confuse the diagnosis.^20^ In the present case, the appearance of the metastasis was not confused with a meningothelial origin (Figure 1).

Simultaneous occurrence of an intracranial meningioma and brain metastases in the same patient at the same time is a rather unusual event. Thus, some thought is needed regarding the pathogenic relationship, pathological diagnosis, surgical indications^10^ and imaging patterns. We conducted a search in the MEDLINE database (using the terms: simultaneous/synchronous, meningioma and metastasis) and only found two papers (Table 1).^10^^,^^21^ Neither of them reported on simultaneous renal cell carcinoma. There are few reports in the literature describing this condition and the largest review on these simultaneous lesions only brought together fifteen cases. Six of them were metastatic lesions, but none of them was from renal cell carcinoma.^9^

**Table 1.**
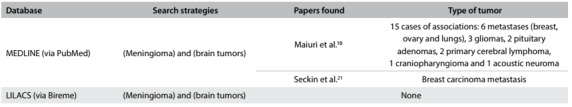
Metastatic brain tumors reported in the literature (PubMed database) as simultaneous presentation with meningiomas

In the case reported here, the patient had a known diagnosis of renal cell carcinoma. Brain MRI showed a ventricular lesion suggestive of metastatic origin. Because the simultaneous olfactory groove meningioma was small at this time, it was preferred to only operate the larger lesion, in order to reduce morbidity that would occur if two different approaches were used. However, after some months, the olfactory groove lesion was found to have increased and a new occipital and symptomatic lesion had appeared.

Neoplasms from the female endocrine and reproductive system are generally more related to meningiomas^10^ and, because of this, are usually present in women. In the present report, however, an even rarer situation was discussed: a male patient with meningioma and brain metastasis, for whom the primary form was renal cell carcinoma. We did not find any reports of this association in the literature review that we conducted.

## CONCLUSIONS

Brain tumors may present through different patterns and, even if they are benign lesions, as meningiomas generally are, they may be associated with rare situations. An occurrence of two brain tumors is one of these situations, and this constitutes a challenge. Simultaneous lesions are an even rarer phenomenon. Metastases are more often reported as part of this entity, although in most cases endocrine and reproductive system tumors have a closer and larger relationship with meningioma growth and therefore are seen more frequently in females. Renal cell carcinoma had not reported until now as part of this association.
